# Peripheral eosinophil trends and clinical outcomes after non-traumatic subarachnoid hemorrhage

**DOI:** 10.3389/fneur.2023.1051732

**Published:** 2023-02-21

**Authors:** Hugo Gonzalez Gomez, Jude P. J. Savarraj, Atzhiry S. Paz, Xuefang Ren, Hua Chen, Louise D. McCullough, Huimahn A. Choi, Aaron M. Gusdon

**Affiliations:** ^1^Division of Neurocritical Care, Department of Neurosurgery, McGovern School of Medicine, University of Texas Health Science Center, Houston, TX, United States; ^2^Department of Neurology, McGovern School of Medicine, University of Texas Health Science Center, Houston, TX, United States

**Keywords:** eosinophils, subarachnoid hemorrhage, inflammation, outcomes, brain injury

## Abstract

**Background/objective:**

Uncontrolled systemic inflammation after non-traumatic subarachnoid hemorrhage (SAH) is associated with worse outcomes. Changes in the peripheral eosinophil count have been linked to worse clinical outcomes after ischemic stroke, intracerebral hemorrhage, and traumatic brain injury. We aimed to investigate the association of eosinophil counts with clinical outcomes after SAH.

**Methods:**

This retrospective observational study included patients with SAH admitted from January 2009 to July 2016. Variables included demographics, modified Fisher scale (mFS), Hunt–Hess Scale (HHS), global cerebral edema (GCE), and the presence of any infection. Peripheral eosinophil counts were examined as part of routine clinical care on admission and daily for 10 days after aneurysmal rupture. Outcome measures included dichotomized discharge mortality, modified Ranked Scale (mRS) score, delayed cerebral ischemia (DCI), vasospasm, and need for ventriculoperitoneal shunt (VPS). Statistical tests included the chi-square test, Student's *t*-test, and multivariable logistic regression (MLR) model.

**Results:**

A total of 451 patients were included. The median age was 54 (IQR 45, 63) years, and 295 (65.4%) were female patients. On admission, 95 patients (21.1%) had a high HHS (>4), and 54 (12.0%) had GCE. A total of 110 (24.4%) patients had angiographic vasospasm, 88 (19.5%) developed DCI, 126 (27.9%) had an infection during hospitalization, and 56 (12.4%) required VPS. Eosinophil counts increased and peaked on days 8–10. Higher eosinophil counts on days 3–5 and day 8 were seen in patients with GCE (*p* < 0.05). Higher eosinophil counts on days 7–9 (*p* < 0.05) occurred in patients with poor discharge functional outcomes. In multivariable logistic regression models, higher day 8 eosinophil count was independently associated with worse discharge mRS (OR 6.72 [95% CI 1.27, 40.4], *p* = 0.03).

**Conclusion:**

This study demonstrated that a delayed increase in eosinophils after SAH occurs and may contribute to functional outcomes. The mechanism of this effect and the relationship with SAH pathophysiology merit further investigation.

## Introduction

Non-traumatic subarachnoid hemorrhage (SAH) is responsible for only 7.2–9.0 hospital admissions per 1,00,000 person/year in the United States; however, it results in significant morbidity and mortality with an estimate of 6,700 annual in-hospital deaths (1, 2). Although recent research has focused on elucidating the pathophysiology of injury after SAH, the mechanisms triggering brain and systemic injury after SAH remain poorly understood. Clinical outcomes range widely; however, several factors have been identified as potential contributors to worse clinical outcomes, including early brain injury (EBI), global cerebral edema (GCE), and delayed cerebral ischemia (DCI) (3).

Initial clinical severity is a strong predictor of the outcome after SAH and drives EBI (4, 5). GCE is a maker of EBI and quantifies the effacement of hemispheric sulci (6). Systemic inflammation is upregulated early after EBI and has been linked to secondary complications such as DCI, which typically occurs 4–14 days after SAH (7, 8). Evidence suggests that inflammation plays an important role in the acute and chronic phases of the disease (9, 10). Initially, aneurysmal rupture and the reaction to extravascular blood trigger the activation of toll-like receptor 4 (TLR4), which sequentially induces an inflammatory response that damages neurons and the white matter (9, 11). The activation of microglia and adhesion molecules within endothelial cells allows inflammatory cells to enter the subarachnoid space. Further release of cytotoxins leads to worsening brain injury correlating with poor clinical outcomes (12–14).

Eosinophils are myeloid cells that have multiple functions, including regulation of the immune response in addition to participation in tissue growth and remodeling (15). Eosinopenia has been associated with poor outcomes in acute cerebral infarction and intracerebral hemorrhage (16, 17). However, eosinophils have been shown to drive the initiation, promotion, and progression of brain tumors such as glioblastoma (18). Although eosinophils are a key part of the inflammatory response, their significance after SAH remains uninvestigated. Eosinophils can release cytotoxic agents such as reactive oxygen species and cytokines and also can release tissue factor from their granules, potentially contributing to clot formation (19, 20). These properties of eosinophils may contribute to increased systemic inflammation and microthrombus formation after aSAH.

The objective of this study was to evaluate the role of eosinophils after SAH and assess associations with clinical outcomes. We hypothesized that increased eosinophil counts after SAH play an important role in the inflammatory cascade and may be associated with poor functional outcomes.

## Patients and methods

### Study population

This was an observational, retrospective, single-center study that included patients with SAH admitted to the Neuroscience Intensive Care Unit at the Memorial Hermann Hospital/University of Texas Health Science Center in Houston from January 2009 to July 2016. Ethical approval was obtained from the Institutional Review Board of the UT Health Science Center. Although data analysis was performed retrospectively, all patients had been prospectively enrolled into an institutional clinical registry, consisting of patients admitted to the neurosciences ICU. Written informed consent had been obtained from the study patients/participants prior to initial enrollment.

### Inclusion and exclusion criteria

Inclusion criteria included a diagnosis of SAH based on CT or the presence of xanthochromia in cerebrospinal fluid (CSF) in patients older than 18 years who were admitted within 48 h of symptoms onset (bleed day 0). Exclusion criteria included traumatic SAH, arteriovenous malformation (AVM), or other non-aneurysmal vascular lesions identified on digital subtraction angiography, the presence of auto-immune diseases, pro-inflammatory conditions like malignancy and pregnancy, and the diagnosis of isolated perimesencephalic SAH. A flowchart depicting the patients selected for analysis is shown in Supplementary Figure 1.

### Demographic, clinical, and radiographic data

On admission, demographic and relevant clinical information was obtained from the electronic medical record. Data collected included Hunt–Hess Scale (HHS), global cerebral edema (GCE), modified Fisher scale (mFS), the presence of intraventricular hemorrhage (IVH), aneurysm location, and the type of treatment. Blood samples for complete blood count (CBC) with differential analysis were collected daily from the day of admission to day 10 of hospitalization to measure peripheral eosinophil count. Clinical outcomes included discharge mortality, functional outcome assessed using the modified Ranking score (mRS), the development of vasospasm, delayed cerebral ischemia (DCI), the presence of any infection (ventriculitis, urinary tract infection, or pneumonia), and the need for ventriculoperitoneal shunt (VPS). All outcome data were adjudicated by at least two attending neurointensivists at a weekly clinical research conference. DCI was defined as the occurrence of a new focal neurological deficit or a decrease of at least two points on the Glasgow Coma Scale lasting for at least 1 h and not attributable to any other causes (10). Vasospasm was defined as any vascular narrowing detected on angiography and did not necessarily need to correlate with a new neurological deficit. Patients were followed after discharge at 3–6 months, and mRS was recorded. All baseline and outcome variables were dichotomized by a functional outcome (mRS) as good (0–3) and bad (4–6).

### Statistical analysis

Statistical tests included the chi-square test for categorical variables and Student's *t*-test for continuous variables. A *p-*value of <0.05 was considered to be statistically significant after false discovery rate correction. Multivariable logistic regression (MLR) models were created to evaluate associations between eosinophil counts and outcomes. Receiver operating characteristic (ROC) curve analysis was performed using the R package pROC. Youden index analysis was performed using the R package cutpointr. All statistical analyses were performed in R (version 4.1.2, R Foundation for Statistical Computing).

## Results

### Baseline characteristics

A total of 451 patients were included in the analysis. Baseline characteristics and outcomes, dichotomized by a discharge mRS score to low (0–3) and high (4–6), are shown in Table 1. The median age was 54 (IQR 45, 63), and 295 (65.4%) were female patients. A total of 221 (49.0%) were treated by coiling, 139 (30.8%) were treated by surgical clipping, and one (0.2%) was treated with a pipeline. No treatment was undertaken in 32 patients (7.1%), and no aneurysm was found in 58 patients (12.9%).

**Table 1:** Demographics and baseline characteristics.

	**Total**	**mRS at discharge (good 0–3)**	**mRS at discharge (bad 4–6)**	***P***
*N*	451	305	146	
Age median (years)^*^	54 (45, 63)	51 (42, 59)	61 (51, 71)	**<0.001**
Sex (female)^†^	295 (65.4)	197 (64.6)	98 (67.1)	0.441
HHS 4–5^†^	95 (21.1)	21 (6.9)	74 (50.7)	**<0.001**
mFS 3–4^†^	307(68.1)	186 (61.0)	121 (82.9)	**<0.001**
GCE^†^	54 (12.0)	24 (7.9)	30 (20.5)	**0.003**
IVH^†^	240 (53.2)	133 (43.6)	107 (73.3)	**<0.001**
**Aneurysm treatment**				
Coiling^†^	221 (49.0)	145 (47.5)	76 (52.1)	0.426
Clipping^†^	139 (30.8)	99 (32.5)	40 (27.4)	0.327
Pipeline^†^	1 (0.2)	1 (0.3)	0 (0)	0.999
No treatment^†^	32 (7.1)	8 (2.6)	24 (16.4)	**<0.001**
No aneurysm found^†^	58 (12.9)	52 (17.0)	6 (4.1)	**<0.001**
**Outcomes**				
Mortality^†^	55 (12.2)	0	55 (37.7)	
Hospital LOS^*^	13 (9, 18)	12 (9, 16)	16 (8, 23)	**0.001**
ICU LOS^*^	10 (7, 14)	10 (7, 12)	13 (8, 17)	**<0.001**
Presence of infection^†^	126 (27.9)	59 (19.3)	67 (45.9)	**<0.001**
DCI^†^	88 (19.5)	39 (12.8)	49 (33.6)	**<0.001**
Angiographic VS^†^	110 (24.4)	61 (20.0)	49 (33.6)	**0.003**
EVD days^*^	9 (7, 12)	8 (7, 11)	10 (8, 14)	**<0.001**
VPS^†^	56 (12.4)	28 (9.2)	28 (19.2)	**0.004**

Data are presented as ^*^median (interquartile range) or^†^N (percent). P-values that are statistically significant are in bold. mRS, modified ranking; GCE, global cerebral edema; IVH, intraventricular hemorrhage; HHS, hunt–hess scale; mFS, modified fisher scale; EVD, external ventricular drain; ICU, intensive care unit; VS, vasospasm; LOS, length of stay; DCI, delayed cerebral ischemia; VPS, ventriculoperitoneal shunt.

Patients with a poor functional outcome were older (*P* < 0.001), had higher HHS (*P* < 0.001), had higher mFS (*P* < 0.001), had a higher prevalence of GCE (*P* = 0.003), and had a higher prevalence of IVH (*P* < 0.001). A higher percentage of patients with a poor functional outcome underwent no treatment for aneurysmal repair (*P* < 0.001), while fewer patients in this group had no aneurysm found (*P* < 0.001). Patients with a worse functional outcome had a higher hospital length of stay (LOS) (*P* = 0.001), higher ICU length of stay (*P* < 0.001), higher rate of infection (*P* < 0.001), higher occurrence of DCI (*P* < 0.001), angiographic vasospasm *(P* = 0.003), longer EVD requirement (*P* < 0.001), and required VPS more commonly (*P* = 0.004).

### Eosinophil count changes and association with disease severity

**Figure 1:**
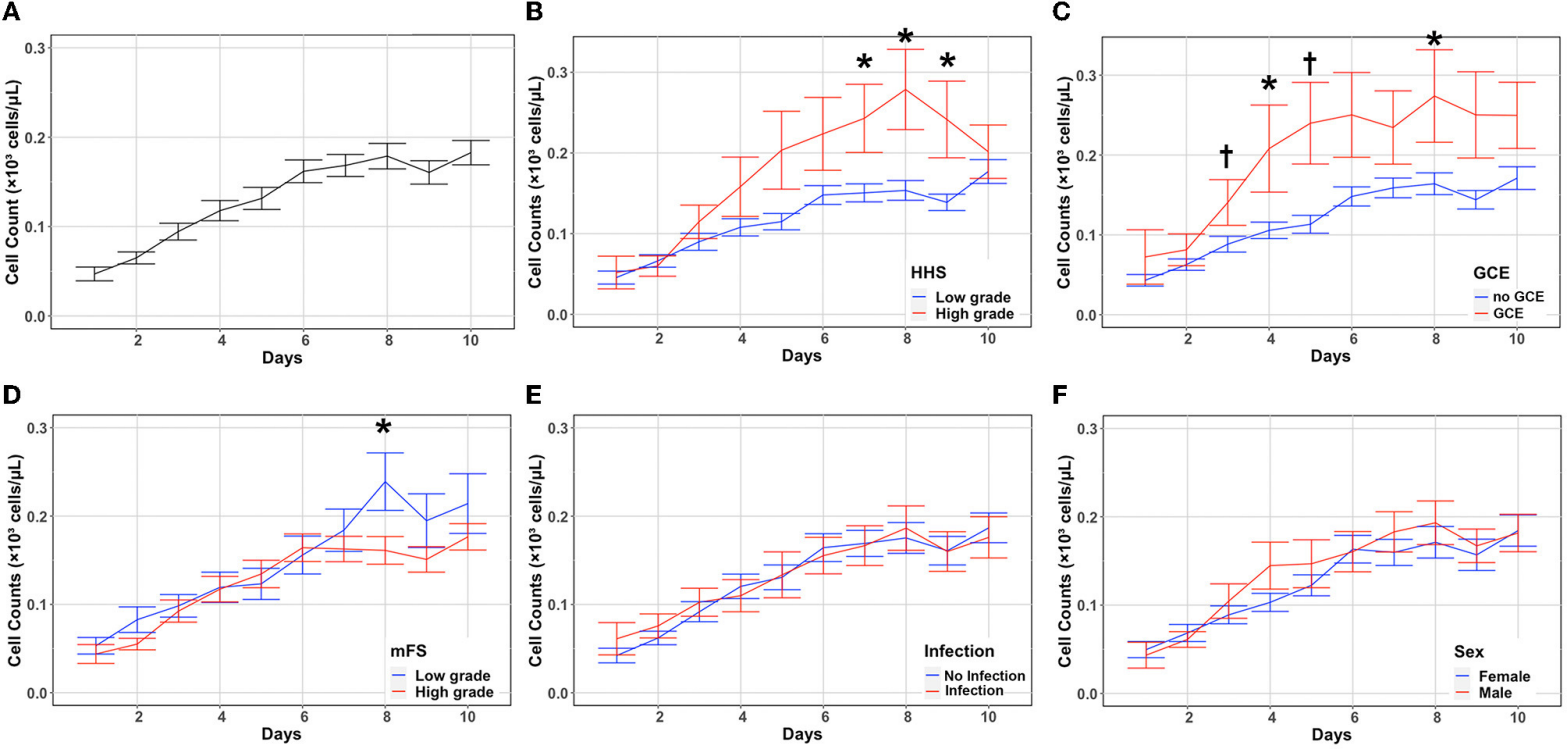
Absolute eosinophil count changes and association with disease severity. Eosinophil counts (mean and standard deviation) are shown from the day of aneurysmal rupture (Day 0) through day 10 **(A)**. Cells are in units of 1,000 per mm^3^. Counts were stratified by HHS **(B)**, GCE **(C)**, mFS **(D)**, infection **(E)**, and sex **(F)**. *P < 0.05, ^†^*P* < 0.001. GCE, global cerebral edema; HHS, hunt–hess scale; mFS, modified fisher scale.

The overall trend in eosinophil count as well as trends stratified according to disease severity (HHS, mFS, and GCE), sex, and the presence of infection during hospitalization are shown in Figure 1. The baseline eosinophil count was 0.04 × 1,000 per mm^3^ after aneurysmal rupture. Eosinophils gradually increased over time and peaked at 0.24 × 1,000 per mm^3^ on day 10 (Figure 1A). Eosinophils were initially similar in patients with higher (HHS 4–5) and lower (HHS 1–3) clinical severity on admission (*P* = 0.073) (Supplementary Table 1). However, in those with higher clinical severity, eosinophils increased more quickly and were significantly higher than in patients with low clinical severity on days 7 (*P* = 0.029), 8 (*P* = 0.017), and 9 (*P* = 0.030) (Figure 1B, Supplementary Table 1). Similarly, patients with and without GCE on admission had a similar eosinophil count initially (*P* = 0.038) (Supplementary Table 2), while those with GCE had significantly higher eosinophil counts on days 3 (*P* = 0.002), 4 (*P* = 0.027), 5 (*P* = 0.002), and 8 (*P* = 0.011) (Figure 1C, Supplementary Table 2). Patients with higher mFS on admission had a higher eosinophil count only on day 8 (*P* = 0.038) (Figure 1D, Supplementary Table 3). There were no significant differences in eosinophil counts when patients were stratified according to the presence of any infection (pneumonia, urinary tract infection, and ventriculitis; Figure 1E, Supplementary Table 4) or sex (Figure 1F, Supplementary Table 5). Daily mean eosinophil values dichotomized by HHS, GCE, mFS, infection, and sex are included in the Supplementary material.

### Eosinophil count changes and associations with SAH outcomes

**Figure 2:**
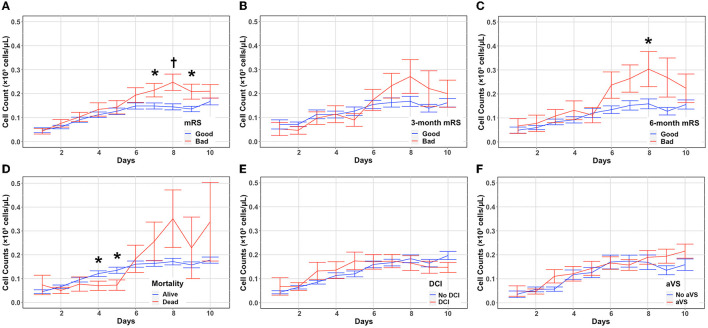
Absolute eosinophil count changes and associations with SAH outcomes. Eosinophil counts (mean and standard deviation) are shown from the day of aneurysmal rupture (Day 0) through day 10. Cells are in units of 1,000 per mm^3^. Counts were stratified by discharge mRS **(A)**, 3-month mRS **(B)**, 6-month mRS **(C)**, in-hospital mortality **(D)**, DCI **(E)**, and aVS **(F)**. *P < 0.05, ^†^*P* < 0.001. aVS, angiographic vasospasm; DCI, delayed cerebral ischemia; mRS, modified Rankin Scale.

A functional outcome was assessed by using the modified Rankin Scale (mRS) at discharge and then during follow up at 3–6 months. Patients with poor mRS (4–6) at discharge had significantly higher eosinophil counts on days 7 (*P* = 0.021), 8 (*P* = 0.0047), and 9 (*P* = 0.0187) (Figure 2A, Supplementary Table 6). Although eosinophil counts were higher in patients with poor mRS at 3 months at later timepoints, it did not reach statistical significance (Figure 2B, Supplementary Table 7). Patients with a worse functional outcome at 6 months had significantly higher eosinophil counts on day 8 (*P* = 0.047) (Figure 2C, Supplementary Table 8). Eosinophils were stratified by discharge mortality, DCI, and angiographic vasospasm. Eosinophil counts were higher in patients without in-hospital morality on days 4 (*P* = 0.048), and 5 (*P* = 0.0303) (Figure 2D, Supplementary Table 9). While there was increased in-hospital mortality in those with higher eosinophil counts on days 7–10, this did not reach statistical significance. No significant differences in eosinophil counts were seen when stratifying according to the occurrence of DCI (Figure 2E, Supplementary Table 10) or angiographic vasospasm (Figure 2F, Supplementary Table 11). Daily mean eosinophil values dichotomized by discharge mRS, 3-month mRS, 6-month mRS, discharge mortality, DCI, and angiographic vasospasm are included in the Supplemental material.

### Outcome models

Univariate models were constructed to assess the relationship between eosinophil count on each day and outcomes (discharge mRS, in-hospital mortality, and DCI) (Supplementary Table 12). Higher day 8 eosinophil count was associated with worse discharge mRS (OR 1.52 [95%CI 1.18–1.99]) and discharge mortality (OR 1.55 [95%CI 1.03, 2.22]) but not DCI (OR 0.93 [95%CI 0.68, 1.21]). Higher day 7 and day 9 eosinophil counts were also associated with worse discharge outcomes; however, there were no associations with mortality or DCI (Supplementary Table 12).

Given these results showing a strong association between higher eosinophils on day 8 and worse clinical outcomes, further analysis was conducted using day 8 eosinophil counts. Demographics between patients with high and low eosinophil count on day 8 were similar, although patients with high eosinophil count tended to have lower mFS but worse discharge mRS (Supplementary Table 13). Multivariable models were created to assess the associations between outcomes and day 8 eosinophil count, accounting for clinically relevant covariables (age, HHS, mFS, GCE, and aneurysm treatment) (Table 2). Elevated day 8 eosinophil count remained significantly associated with poor discharge mRS after correction for covariables (OR 6.72 [95% CI 1.27, 40.4], *P* = 0.0301). At day 8, eosinophil counts were not significantly associated with in-hospital mortality (OR 1.86 [95% CI 0.68, 108.9], *P* = 0.105) or DCI (OR 0.59 [95% CI 0.12, 2.66], *P* = 0.508) after correction for covariables. Older age was associated with worse discharge mRS (OR 1.07 [95% CI 1.04, 1.10], *P* = 5.91 × 10^−6^) and increased discharge mortality (OR 1.12 [95% CI 1.02, 1.27], *P* = 0.033). Similarly, higher HHS on admission was associated with poor discharge mRS (OR 7.50 [95% CI 3.36, 17.8], *P* = 1.85 × 10^−6^) and increased in-hospital mortality (OR 13.3 [95% CI 1.81, 162.3], *P* = 0.019). The presence of GCE on admission (OR 2.64 [95% CI 1.11, 6.26], *P* = 0.0268) and aneurysm treatment by clipping (OR 5.55 [3.06, 9.35], *P* = 0.0334) was associated with the development of DCI.

**Table 2:** Multivariable models for clinical outcomes based on day 8.

	**Discharge mRS**		**Discharge Mortality**		**DCI**	
**Variable**	**OR (CI)**	***p***	**OR (CI)**	***p***	**OR (CI)**	***p***
Day 8 eosinophils	1.46 (1.05, 2.10)	**0.030**	1.13 (0.93, 2.56)	0.105	0.90 (0.65, 1.22)	0.508
Age	1.07 (1.04, 1.10)	**5.91** **×10^−6^**	1.12 (1.02, 1.27)	**0.033**	0.99 (0.97, 1.02)	0.939
Sex (male)	1.23 (0.95, 2.26)	0.578	6.04 (5.28, 25.9)	**0.0069**	1.09 (0.55, 2.15)	0.795
HHS	7.50 (3.36, 17.8)	**1.85** **×10^−6^**	13.3 (1.81, 16.2)	**0.019**	1.59 (0.72, 3.42)	0.237
mFS	1.59 (0.63, 4.35)	0.344	13.4 (0.97, 4.84)	0.092	2.06 (0.78, 6.50)	0.173
GCE	1.48 (0.52, 4.07)	0.455	0.82 (0.28, 9.25)	0.075	2.64 (1.11, 6.26)	**0.0268**
Aneurysm clipping	0.67 (0.38, 1.14)	0.147	5.61 (1.57, 2.9)	**0.015**	5.55 (3.06, 9.35)	**0.0334**

Data are presented as OR [95%CI (P-value)]. P-values that are statistically significant are in bold.

OR, odds ratio; CI, 95% confidence interval; GCS, glasgow coma scale; mRS, modified rankin scale; DCI, delayed cerebral ischemia; HHS, hunt–hess scale; mFS, modified fisher scale; GCE, global cerebral edema. OR is represented as per 0.2 × 10^3^ cell/μL increase in eosinophil.

ROC analysis was performed to determine the ability of day 8 eosinophil count to predict outcomes. Day 8 eosinophil had an area under the curve (AUC) of 0.775 (95% CI 0.708, 0.842). Youden index analysis was performed to determine the optimal cutoff of day 8 eosinophil. An eosinophil value of 0.191 × 10^3^ cells/μl predicted discharge mRS (0–3 vs. 4–6) with a sensitivity of 0.690 and specificity of 0.750 (Supplementary Figure 3).

## Discussion

Although the peripheral inflammatory response to neurological injury has been the subject of much recent research, the role of peripheral eosinophils has been relatively understudied. This is the first study to demonstrate an association between eosinophil counts and outcomes after SAH. Herein, we have shown that although eosinophil counts are normal at the time of admission, eosinophils increase gradually after an aneurysmal rupture with a more rapid increase seen in those patients with higher clinical severity and GCE. Eosinophil counts later after injury, notably on the post-bleed day (PBD) 8, had an independent association with functional outcomes. PBD 8 eosinophil count also had moderate sensitivity and high specificity for predicting poor outcomes.

Although to our knowledge these are the first data to be reported in SAH, changes in peripheral eosinophils have been linked to poor functional outcomes in other acute brain injuries. In acute ischemic stroke, early eosinopenia has been associated with larger infarct volume and a higher rate of infection (21). Previous studies have also shown that early eosinopenia can be an independent predictor of severity, poor outcome, and increased mortality in acute ischemic stroke (17, 22, 23). A variety of ratios of eosinophils to other leukocytes including monocytes and neutrophils have also been studied, with lower eosinophil counts typically correlating with poor outcomes (24–26). Similarly, early eosinopenia after intracerebral hemorrhage (ICH) has been associated with increased mortality (16). Eosinopenia on admission was also shown to be associated with poor functional outcomes and longer hospital stays in pediatric patients with traumatic brain injury (27). It has been suggested that eosinopenia after brain injury may be reflective of peripheral immunosuppression contributing to an increased risk of infection (17) although other mechanisms may also contribute.

However, the deleterious effects of increased eosinophil counts have also been reported. One study found an association between increased eosinophils on admission and an increased risk of hematoma expansion after ICH (28). Increased eosinophil counts have also been associated with the development of complex aortic plaques, potentially mediated by the effects of eosinophil cationic protein (29, 30). Hypereosinophilia has also been linked to multisystem organ failure and ischemic strokes (31). Eosinophils are thought to contribute to thrombotic events by releasing a tissue factor stored in their granules (32). The release of cytotoxic substances such as reactive oxygen species and cytokines may also play a role in the deleterious effects mediated by eosinophils (19).

As our study is the first one to investigate the impact of eosinophil counts after SAH, a direct comparison with the available literature on non-traumatic SAH outcomes cannot be conducted. Although there are reported cases and case series of hypereosinophilic syndromes and subarachnoid hemorrhage (33–35), no large studies pertaining to eosinophil count and SAH outcomes have been published. Contrary to the available evidence of eosinophils in other acute neurological diseases, our results demonstrated a correlation between higher eosinophil counts, particularly on PBD 8, and a poor functional outcome after non-traumatic SAH. A key difference between this study and previous literature is our assessment of eosinophil count over 10 days after injury. Indeed, although significant differences in eosinophil count were not seen early after injury, higher eosinophil counts were seen in those patients with higher clinical severity and poor outcomes several days after aneurysmal rupture. Furthermore, although previous literature has suggested the potential role of eosinophils in mitigating the risk of infection, we saw no significant difference in eosinophil count comparing those patients with or without infections during the course of hospitalization (Figure 1E), suggesting that eosinophils play a role after SAH independent of infection.

The mechanisms linking increased eosinophil counts on post-bleeding day 8 to poor outcomes remain unclear. However, we speculate that eosinophils may interact with the systemic inflammatory response occurring after non-traumatic SAH. Previous studies have shown that systemic inflammation peaks are seen at 24–48 h after SAH (36). Cytokines, such as CCL5 (RANTES), act as chemoattractants for a variety of inflammatory cells including eosinophils (37, 38). It is possible that the delayed increase in eosinophils reported herein is in response to systemic inflammation triggered by an aneurysmal rupture. We also show that patients with increased GCE on admission had increased eosinophil counts starting on post-bleed day 3 (Figure 1D). Cerebral edema in SAH is a complex and multifactorial process, caused by early ischemia, dysfunction of cerebral autoregulation, blood product decomposition, endocrine abnormalities, and neuroinflammation (39). CNS inflammation is able to regulate peripheral leukocytes by the shedding of astrocyte-derived extracellular vesicles (40).

While the mechanisms linking GCE and increased systemic eosinophil counts need to be elucidated, it is tempting to speculate that crosstalk between the CNS and peripheral inflammatory responses drives delayed increases in eosinophils. In other neuroinflammatory conditions, particularly neuromyelitis optica (NMO), eosinophils have been shown to infiltrate into the CNS to the location of active lesions (41, 42). Mice deficient in eosinophils have been shown to have less severe NMO lesions (43). Additional basic science studies will be required to determine whether systemic eosinophils also migrate to the brain after SAH. Although we did not find an association between eosinophil count and DCI in this study, it is possible that the pro-inflammatory and prothrombotic milieu created by eosinophils could affect the pathophysiology contributing to DCI.

Interestingly, subgroup analyses suggested that the association between eosinophils and outcomes was most prominent in younger (<55 years of age) but not older (≥55 years of age) patients (Supplementary Figure 2). Systemic eosinophils have been shown to decrease with age (44). An eosinophil function has also been shown to be affected by age, with decreased degranulation and free radical production in response to stimulation in older patients (45). Although limited by a smaller sample size, this subgroup analysis may support a diminished inflammatory role of eosinophils after SAH in older patients.

This study has several important limitations. A major caveat is the use of corticosteroids in this patient population. During the period of the study, our institution had a protocol to treat patients with non-traumatic SAH that included a standard regimen of corticosteroids for 1 week after the ictus. Corticosteroids have important anti-inflammatory effects including the inhibition of inflammatory cells and decreasing expression of adhesion molecules (46). In addition, steroids induce eosinophil clearance by increasing apoptosis and inhibiting survivability pathways induced by cytokines such as IL-3, IL-5, and GM-CSF (47, 48). Compared to other immune system cells, steroids appear to have less of an effect on eosinophils. Although we cannot discount the effect of corticosteroids, all patients were treated with the same dosing and duration. Another limitation of our study is that our results were not adjusted to include the potential effect of medication-related drug reactions. These reactions are a cause of elevation of peripheral eosinophils because of type IVb reactions, involving the Th2-mediated immune response and secretion of cytokines such as IL-4, IL-13, and IL-5 and also B-cell production of IgE (49). This study was also based on the results from a single tertiary center, thus potentially resulting in selection bias and limiting generalizability. Although we have shown independent associations between eosinophil counts and outcomes, we are limited in our ability to draw a mechanistic conclusion. Future studies will be required to assess the interactions between the systemic cytokine response and eosinophils as well as the role and mechanism of peripheral eosinophilia in driving neuroinflammation.

## Conclusion

To our knowledge, this is the first study to assess the role of eosinophils after non-traumatic SAH. We demonstrated that a delayed increase in eosinophils after SAH may contribute to functional outcomes. The mechanism of this effect as well as the relationship with cerebral edema merit further investigation.

## Data availability statement

The raw data supporting the conclusions of this article will be made available by the authors, without undue reservation.

## Ethics statement

The studies involving human participants were reviewed and approved by University of Texas Institutional Review Board. Written informed consent for participation was not required for this study in accordance with the national legislation and the institutional requirements.

## Author contributions

HG and AG designed the study, analyzed data, and wrote and revised the manuscript. JS analyzed data and revised the manuscript. AP and HC acquired data and revised the manuscript. XR analyzed data and edited the manuscript. HAC contributed to the study design and edited the manuscript. All authors contributed to the article and approved the submitted version.
