# Supplementary material for: Peripheral eosinophil trends and clinical outcomes after non-traumatic subarachnoid hemorrhage
Source: Front Neurol. 2023 Feb 21;14:1051732. doi: 10.3389/fneur.2023.1051732 (PMC9989180; doi:10.3389/fneur.2023.1051732)
Supplement: Supplementary file 1 [file Table_1.DOCX]

**Supplementary Material**

1. Supplementary Figure 1: Flowchart depicting patient selection
2. Supplementary Figure 2: Forest plot for univariate models considering the association between day 8 eosinophil count and discharge mRS across patient subgroups.
3. Supplementary Figure 3: ROC curve for the ability of day 8 eosinophil count to predict outcomes
4. Supplementary Table 1: Daily Values for Low vs High Hunt Hess Scale Score
5. Supplementary Table 2: Daily Values for Global Cerebral Edema
6. Supplementary Table 3: Daily Values for Modified Fischer Scale (mFS) Score
7. Supplementary Table 4: Daily Values for Infection
8. Supplementary Table 5: Daily Values for Sex
9. Supplementary Table 6: Daily Values for Discharge mRS Outcomes
10. Supplementary Table 7: Daily Values for 3-month mRS Outcomes
11. Supplementary Table 8: Daily Values for 6-month mRS Outcomes
12. Supplementary Table 9: Daily Values for Mortality
13. Supplementary Table 10: Daily Values for DCI
14. Supplementary Table 11: Daily Values for Angiographic Vasospasm
15. Supplementary Table 12: Univariable Models for eosinophil counts from each day
16. Supplementary Table 13: Demographics and baseline characteristics based on high or low day 8 eosinophil count

**
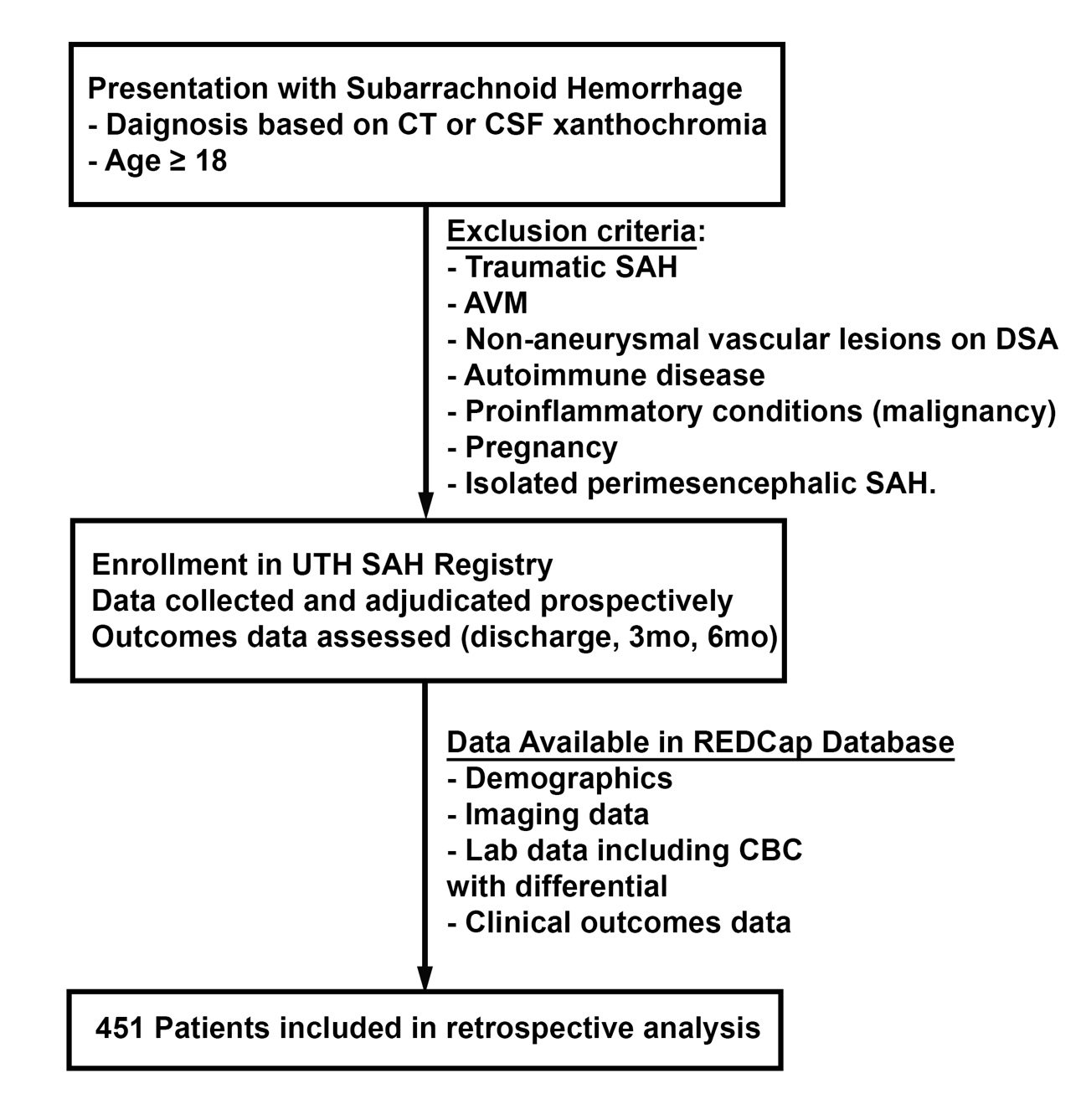
**

**Supplementary Figure 1: Flowchart depicting patient enrollment.** Abbreviations: subarachnoid hemorrhage (SAH), computed tomography (CT), cerebrospinal fluid (CSF), arteriovenous malformation (AVM), digital subtraction angiography (DSA), University of Texas Health Science Center at Houston (UTH), mo (month), completed blood count (CBC)

**
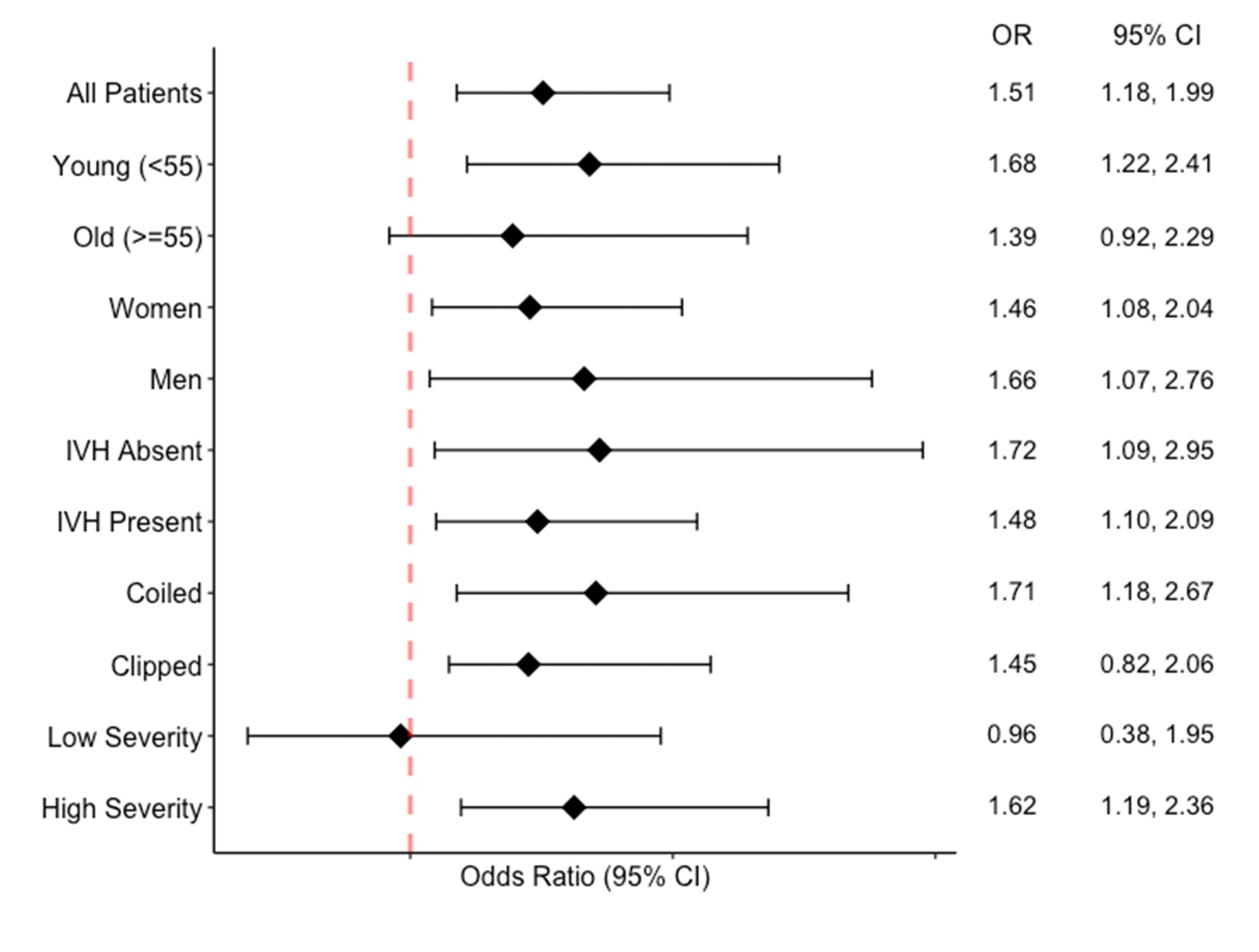
**

**Supplementary Figure 2: Forest plot for univariate models considering the association between day 8 eosinophil count and discharge mRS across patient subgroups.** Univariate models were created to assess the association between day 8 eosinophil count and discharge mRS (4-6). Low severity indicated Hunt Hess Scale ≤3, while high severity indicates Hunt Hess Scale ≥4. All odds ratios are presented as odds per 0.2 x10^3^ cell/µL change in eosinophil count. Abbreviations: odds ratio (OR), confidence interval (CI), intraventricular hemorrhage (IVH).

**
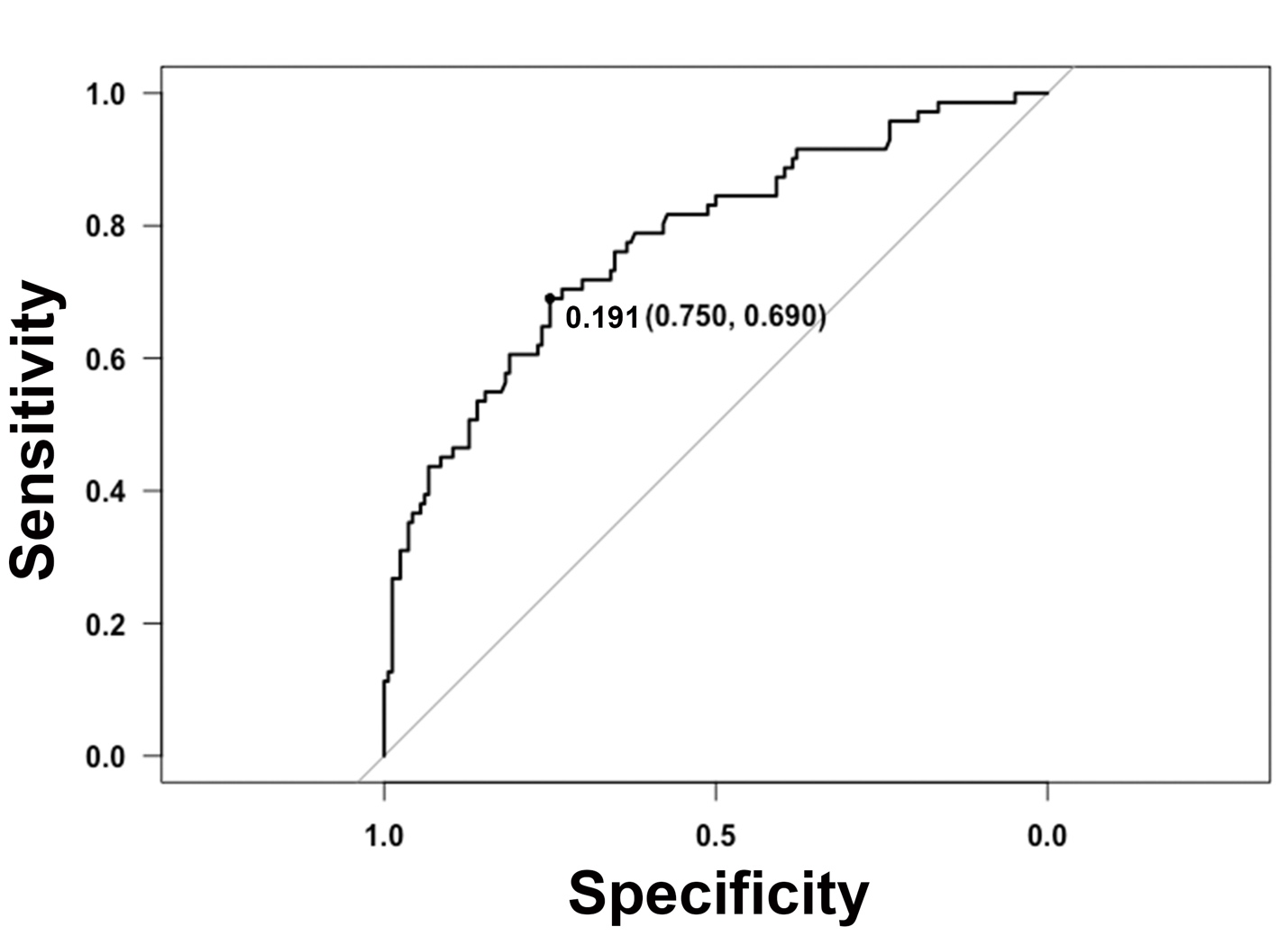
**

**Supplementary Figure 3: ROC curve for the ability of day 8 eosinophil count to predict outcomes.** Day 8 eosinophil count was used to predict discharge modified Rankin Scale (mRS). The optimal Youden index is displayed with corresponding sensitivity and specificity.

**Supplementary Table 1:** **Daily Values for Low vs High Hunt Hess Scale Score**

|  | **Low HHS** | | | **High HHS** | | |  |
| --- | --- | --- | --- | --- | --- | --- | --- |
| **Eosinophil Count** | **Mean** | **St. Dev** | **N** | **Mean** | **St. Dev** | **N** | **P-Value** |
| **Day 1** | 0.0442 | 0.0996 | 91 | 0.0518 | 0.1329 | 99 | 0.7311 |
| **Day 2** | 0.0643 | 0.0927 | 102 | 0.0598 | 0.0778 | 102 | 0.7643 |
| **Day 3** | 0.0863 | 0.1425 | 115 | 0.1146 | 0.1328 | 112 | 0.2319 |
| **Day 4** | 0.1006 | 0.1404 | 114 | 0.1581 | 0.2523 | 123 | 0.1389 |
| **Day 5** | 0.1100 | 0.1363 | 116 | 0.2034 | 0.3308 | 134 | 0.0634 |
| **Day 6** | 0.1428 | 0.1666 | 116 | 0.2237 | 0.3129 | 146 | 0.0889 |
| **Day 7** | 0.1452 | 0.1596 | 119 | 0.2428 | 0.3048 | 151 | **0.0293** |
| **Day 8** | 0.1520 | 0.1697 | 97 | 0.2787 | 0.3442 | 142 | **0.0167** |
| **Day 9** | 0.1332 | 0.1320 | 89 | 0.2415 | 0.3325 | 142 | **0.0300** |
| **Day 10** | 0.1760 | 0.1874 | 74 | 0.2015 | 0.2316 | 136 | 0.4844 |

Abbreviations: Hunt Hess Scale (HHS), standard deviation (St. Dev.)

**Supplementary Table 2: Daily Values for Global Cerebral Edema**

|  | **Low GCE** | | | **High GCE** | | |  |
| --- | --- | --- | --- | --- | --- | --- | --- |
| **Eosinophil Count** | **Mean** | **St. Dev.** | **N** | **Mean** | **St. Dev.** | **N** | **P-Value** |
| **Day 1** | 0.0416 | 0.0943 | 165 | 0.0723 | 0.1703 | 25 | 0.3876 |
| **Day 2** | 0.0608 | 0.0886 | 180 | 0.0813 | 0.0975 | 24 | 0.1452 |
| **Day 3** | 0.0850 | 0.1391 | 201 | 0.1406 | 0.1459 | 26 | **0.0020** |
| **Day 4** | 0.0990 | 0.1429 | 209 | 0.2081 | 0.2885 | 28 | **0.0270** |
| **Day 5** | 0.1086 | 0.1583 | 214 | 0.2399 | 0.3066 | 36 | **0.0021** |
| **Day 6** | 0.1434 | 0.1773 | 227 | 0.2503 | 0.3139 | 35 | 0.0568 |
| **Day 7** | 0.1542 | 0.1869 | 236 | 0.2345 | 0.2675 | 34 | 0.0996 |
| **Day 8** | 0.1630 | 0.1979 | 207 | 0.2739 | 0.3276 | 32 | **0.0402** |
| **Day 9** | 0.1390 | 0.1590 | 195 | 0.2503 | 0.3240 | 36 | 0.0509 |
| **Day 10** | 0.1702 | 0.1904 | 179 | 0.2497 | 0.2307 | 31 | 0.0778 |

Abbreviations: global cerebral edema (GCE), standard deviation (St. Dev.)

**Supplementary Table 3: Daily Values for Modified Fisher Scale (mFS) Score**

|  | **Low mFS** | | | **High mFS** | | |  |
| --- | --- | --- | --- | --- | --- | --- | --- |
| **Eosinophil Count** | **Mean** | **St. Dev.** | **N** | **Mean** | **St. Dev.** | **N** | **P-Value** |
| **Day 1** | 0.0521 | 0.0737 | 65 | 0.0438 | 0.1194 | 125 | 0.5825 |
| **Day 2** | 0.0822 | 0.1153 | 73 | 0.0550 | 0.0747 | 131 | 0.1037 |
| **Day 3** | 0.0902 | 0.0948 | 74 | 0.0924 | 0.1552 | 153 | 0.9006 |
| **Day 4** | 0.1001 | 0.1265 | 73 | 0.1172 | 0.1848 | 164 | 0.4332 |
| **Day 5** | 0.1078 | 0.1120 | 65 | 0.1344 | 0.2117 | 185 | 0.2132 |
| **Day 6** | 0.1408 | 0.1758 | 74 | 0.1641 | 0.2134 | 188 | 0.3880 |
| **Day 7** | 0.1711 | 0.1980 | 73 | 0.1627 | 0.2017 | 197 | 0.7708 |
| **Day 8** | 0.2458 | 0.2514 | 55 | 0.1611 | 0.2122 | 184 | **0.0375** |
| **Day 9** | 0.1794 | 0.2173 | 51 | 0.1509 | 0.1930 | 180 | 0.4207 |
| **Day 10** | 0.2119 | 0.2044 | 35 | 0.1764 | 0.1973 | 175 | 0.3631 |

Abbreviations: modified Fischer Scale (mFS), standard deviation (St. Dev.)

**Supplementary Table 4: Daily Values for Infection**

|  | **No Infection** | | | **Infection** | | |  |
| --- | --- | --- | --- | --- | --- | --- | --- |
| **Eosinophil Count** | **Mean** | **St. Dev** | **N** | **Mean** | **St. Dev** | **N** | **P-Value** |
| **Day 1** | 0.0403 | 0.1006 | 142 | 0.0612 | 0.1266 | 48 | 0.3087 |
| **Day 2** | 0.0597 | 0.0899 | 161 | 0.0757 | 0.0891 | 43 | 0.3063 |
| **Day 3** | 0.0881 | 0.1483 | 173 | 0.1025 | 0.1167 | 54 | 0.4692 |
| **Day 4** | 0.1135 | 0.1808 | 177 | 0.1099 | 0.1412 | 60 | 0.8763 |
| **Day 5** | 0.1258 | 0.1840 | 182 | 0.1335 | 0.2144 | 68 | 0.7931 |
| **Day 6** | 0.1594 | 0.2152 | 189 | 0.1553 | 0.1765 | 73 | 0.8761 |
| **Day 7** | 0.1639 | 0.2006 | 190 | 0.1666 | 0.2014 | 80 | 0.9199 |
| **Day 8** | 0.1745 | 0.2264 | 165 | 0.1865 | 0.2162 | 74 | 0.7005 |
| **Day 9** | 0.1550 | 0.1986 | 153 | 0.1599 | 0.1982 | 78 | 0.8579 |
| **Day 10** | 0.1858 | 0.1935 | 131 | 0.1760 | 0.2072 | 79 | 0.7343 |

Abbreviations: modified Rankin Scale (mRS), standard deviation (St. Dev.)

**Supplementary Table 5: Daily Values for Sex**

|  | **Male** | | | **Female** | | |  |
| --- | --- | --- | --- | --- | --- | --- | --- |
| **Eosinophil Count** | **Mean** | **St. Dev.** | **N** | **Mean** | **St. Dev.** | **N** | **P-Value** |
| **Day 1** | 0.0432 | 0.1250 | 70 | 0.0469 | 0.0987 | 120 | 0.8416 |
| **Day 2** | 0.0631 | 0.0786 | 76 | 0.0637 | 0.0966 | 128 | 0.9633 |
| **Day 3** | 0.1080 | 0.1809 | 82 | 0.0828 | 0.1127 | 145 | 0.2730 |
| **Day 4** | 0.1412 | 0.2376 | 81 | 0.0971 | 0.1213 | 158 | 0.1296 |
| **Day 5** | 0.1418 | 0.2553 | 92 | 0.1201 | 0.1460 | 160 | 0.4641 |
| **Day 6** | 0.1582 | 0.2197 | 92 | 0.1593 | 0.1976 | 171 | 0.9691 |
| **Day 7** | 0.1847 | 0.2201 | 91 | 0.1548 | 0.1903 | 181 | 0.2796 |
| **Day 8** | 0.1922 | 0.2199 | 78 | 0.1714 | 0.2255 | 164 | 0.5074 |
| **Day 9** | 0.1637 | 0.1701 | 92 | 0.1531 | 0.2132 | 152 | 0.6857 |
| **Day 10** | 0.1820 | 0.1799 | 71 | 0.1832 | 0.2081 | 144 | 0.9662 |

Abbreviations: standard deviation (St. Dev.)

**Supplementary Table 6: Daily Values for Discharge mRS Outcomes**

|  | **Good Discharge Outcome** | | | **Bad Discharge Outcome** | | |  |
| --- | --- | --- | --- | --- | --- | --- | --- |
| **Eosinophil Count** | **Mean** | **St. Dev.** | **N** | **Mean** | **St. Dev.** | **N** | **P-Value** |
| **Day 1** | 0.0472 | 0.1175 | 136 | 0.0436 | 0.0862 | 54 | 0.8235 |
| **Day 2** | 0.0586 | 0.0794 | 153 | 0.0764 | 0.1131 | 51 | 0.3030 |
| **Day 3** | 0.0876 | 0.1384 | 169 | 0.1031 | 0.1475 | 58 | 0.4885 |
| **Day 4** | 0.1044 | 0.1487 | 175 | 0.1335 | 0.2178 | 62 | 0.3329 |
| **Day 5** | 0.1216 | 0.1733 | 179 | 0.1434 | 0.2335 | 71 | 0.4798 |
| **Day 6** | 0.1430 | 0.1717 | 187 | 0.1942 | 0.2641 | 75 | 0.1250 |
| **Day 7** | 0.1426 | 0.1696 | 190 | 0.2146 | 0.2508 | 80 | **0.0210** |
| **Day 8** | 0.1429 | 0.1609 | 161 | 0.2479 | 0.2996 | 78 | **0.0047** |
| **Day 9** | 0.1294 | 0.1346 | 153 | 0.2085 | 0.2754 | 78 | **0.0187** |
| **Day 10** | 0.1666 | 0.1678 | 136 | 0.2101 | 0.2430 | 74 | 0.1733 |

Abbreviations: modified Rankin Scale (mRS), standard deviation (St. Dev.)

**Supplementary Table 7: Daily Values for 3-month mRS Outcomes**

|  | **Good 3-month Outcome** | | | **Bad 3-month Outcome** | | |  |
| --- | --- | --- | --- | --- | --- | --- | --- |
| **Eosinophil Count** | **Mean** | **St. Dev.** | **N** | **Mean** | **St. Dev.** | **N** | **P-Value** |
| **Day 1** | 0.0714 | 0.1535 | 156 | 0.0526 | 0.1257 | 34 | 0.5767 |
| **Day 2** | 0.0719 | 0.0860 | 170 | 0.0468 | 0.0625 | 44 | 0.1932 |
| **Day 3** | 0.1135 | 0.1755 | 184 | 0.0975 | 0.1046 | 43 | 0.6199 |
| **Day 4** | 0.1125 | 0.1439 | 190 | 0.1140 | 0.1527 | 47 | 0.9687 |
| **Day 5** | 0.1264 | 0.1807 | 199 | 0.0899 | 0.1260 | 51 | 0.2547 |
| **Day 6** | 0.1547 | 0.1813 | 207 | 0.1732 | 0.2151 | 55 | 0.6974 |
| **Day 7** | 0.1628 | 0.2049 | 205 | 0.2327 | 0.2570 | 65 | 0.2122 |
| **Day 8** | 0.1676 | 0.2041 | 180 | 0.2710 | 0.3380 | 59 | 0.1717 |
| **Day 9** | 0.1391 | 0.1576 | 171 | 0.2212 | 0.3471 | 60 | 0.2895 |
| **Day 10** | 0.1616 | 0.1743 | 156 | 0.1995 | 0.2712 | 54 | 0.5286 |

Abbreviations: modified Rankin Scale (mRS), standard deviation (St. Dev.)

**Supplementary Table 8: Daily Values for 6-month mRS Outcomes**

|  | **Good 6-month Outcome** | | | **Bad 6-month Outcome** | | |  |
| --- | --- | --- | --- | --- | --- | --- | --- |
| **Eosinophil Count** | **Mean** | **St. Dev.** | **N** | **Mean** | **St. Dev.** | **N** | **P-Value** |
| **Day 1** | 0.0489 | 0.0980 | 58 | 0.0665 | 0.1350 | 18 | 0.6058 |
| **Day 2** | 0.0597 | 0.0751 | 72 | 0.0773 | 0.1372 | 14 | 0.6264 |
| **Day 3** | 0.0834 | 0.1068 | 82 | 0.1078 | 0.1050 | 13 | 0.4191 |
| **Day 4** | 0.0922 | 0.1236 | 87 | 0.1317 | 0.1800 | 19 | 0.3486 |
| **Day 5** | 0.1204 | 0.1804 | 87 | 0.1116 | 0.1516 | 21 | 0.8128 |
| **Day 6** | 0.1344 | 0.1526 | 92 | 0.2370 | 0.2685 | 23 | 0.0833 |
| **Day 7** | 0.1522 | 0.1881 | 92 | 0.2642 | 0.2841 | 24 | 0.0721 |
| **Day 8** | 0.1588 | 0.1195 | 76 | 0.3035 | 0.2171 | 23 | **0.0467** |
| **Day 9** | 0.1280 | 0.1379 | 76 | 0.2684 | 0.3822 | 21 | 0.1045 |
| **Day 10** | 0.1560 | 0.1681 | 77 | 0.2237 | 0.2790 | 21 | 0.2885 |

Abbreviations: modified Rankin Scale (mRS), standard deviation (St. Dev.)

**Supplementary Table 9: Daily Values for Mortality**

|  | **No Mortality** | | | **Mortality** | | |  |
| --- | --- | --- | --- | --- | --- | --- | --- |
| **Eosinophil Count** | **Mean** | **St. Dev.** | **N** | **Mean** | **St. Dev.** | **N** | **P-Value** |
| **Day 1** | 0.0437 | 0.1041 | 176 | 0.0732 | 0.1519 | 14 | 0.4882 |
| **Day 2** | 0.0642 | 0.0919 | 193 | 0.0500 | 0.0433 | 11 | 0.3507 |
| **Day 3** | 0.0927 | 0.1429 | 215 | 0.0768 | 0.1037 | 12 | 0.6219 |
| **Day 4** | 0.1154 | 0.1753 | 224 | 0.0706 | 0.0671 | 13 | **0.0479** |
| **Day 5** | 0.1311 | 0.1966 | 237 | 0.0724 | 0.0784 | 13 | **0.0303** |
| **Day 6** | 0.1567 | 0.2037 | 247 | 0.1827 | 0.2216 | 15 | 0.6633 |
| **Day 7** | 0.1595 | 0.1926 | 256 | 0.2560 | 0.3038 | 14 | 0.2601 |
| **Day 8** | 0.1705 | 0.2110 | 229 | 0.3514 | 0.3820 | 10 | 0.1702 |
| **Day 9** | 0.1533 | 0.1839 | 221 | 0.2290 | 0.4084 | 10 | 0.5737 |
| **Day 10** | 0.1766 | 0.1847 | 203 | 0.3386 | 0.4369 | 7 | 0.3653 |

Abbreviations: standard deviation (St. Dev.)

**Supplementary Table 10: Daily Values for DCI**

|  | **No DCI** | | | **DCI** | | |  |
| --- | --- | --- | --- | --- | --- | --- | --- |
| **Eosinophil Count** | **Mean** | **St. Dev.** | **N** | **Mean** | **St. Dev.** | **N** | **P-Value** |
| **Day 1** | 0.0419 | 0.0841 | 162 | 0.0676 | 0.1914 | 28 | 0.4919 |
| **Day 2** | 0.0625 | 0.0893 | 171 | 0.0674 | 0.0929 | 33 | 0.7849 |
| **Day 3** | 0.0816 | 0.1015 | 184 | 0.1321 | 0.2373 | 43 | 0.1788 |
| **Day 4** | 0.1066 | 0.1501 | 191 | 0.1358 | 0.2357 | 46 | 0.4265 |
| **Day 5** | 0.1151 | 0.1642 | 197 | 0.1741 | 0.2681 | 53 | 0.1328 |
| **Day 6** | 0.1547 | 0.1969 | 205 | 0.1701 | 0.2297 | 57 | 0.6476 |
| **Day 7** | 0.1622 | 0.1975 | 211 | 0.1732 | 0.2118 | 59 | 0.7239 |
| **Day 8** | 0.1828 | 0.2200 | 181 | 0.1651 | 0.2325 | 58 | 0.6132 |
| **Day 9** | 0.1598 | 0.2015 | 174 | 0.1476 | 0.1888 | 59 | 0.6792 |
| **Day 10** | 0.1959 | 0.2106 | 151 | 0.1472 | 0.1597 | 59 | 0.0736 |

Abbreviations: delayed cerebral ischemia (DCI), standard deviation (St. Dev.)

**Supplementary Table 11: Daily Values for Angiographic Vasospasm**

|  | **No Vasospasm** | | | **Vasospasm** | | |  |
| --- | --- | --- | --- | --- | --- | --- | --- |
| **Eosinophil Count** | **Mean** | **St. Dev.** | **N** | **Mean** | **St. Dev.** | **N** | **P-Value** |
| **Day 1** | 0.0354 | 0.0906 | 122 | 0.0480 | 0.1483 | 68 | 0.6292 |
| **Day 2** | 0.0557 | 0.0645 | 131 | 0.0477 | 0.0731 | 73 | 0.5928 |
| **Day 3** | 0.0567 | 0.0590 | 144 | 0.1082 | 0.2147 | 83 | 0.0867 |
| **Day 4** | 0.1171 | 0.1401 | 151 | 0.1182 | 0.2383 | 86 | 0.9768 |
| **Day 5** | 0.1259 | 0.1596 | 157 | 0.1432 | 0.2464 | 93 | 0.6299 |
| **Day 6** | 0.1699 | 0.2172 | 165 | 0.1657 | 0.2264 | 97 | 0.9137 |
| **Day 7** | 0.1735 | 0.1951 | 171 | 0.1574 | 0.1952 | 99 | 0.6308 |
| **Day 8** | 0.1688 | 0.2085 | 141 | 0.1894 | 0.2557 | 98 | 0.6222 |
| **Day 9** | 0.1355 | 0.1257 | 134 | 0.1941 | 0.2558 | 99 | 0.0940 |
| **Day 10** | 0.1587 | 0.1515 | 111 | 0.2152 | 0.2492 | 99 | 0.1387 |

Abbreviations: standard deviation (St. Dev.)

**Supplementary Table 12:** Univariable models for eosinophil counts from each day

| **Eosinophil Count** | **Discharge mRS** | **Mortality** | **DCI** |
| --- | --- | --- | --- |
| **Day 1** | -0.32±1.58, *P*=0.84* | 1.64±1.76, *P*=0.35 | 1.63±1.51, *P*=0.28 |
| **Day 2** | 2.05±1.71, *P*=0.23 | -2.18±4.30, *P*=0.61 | 0.58±2.06, *P*=0.78 |
| **Day 3** | 0.72±1.02, *P*=0.48 | -1.06±2.78, *P*=0.70 | 2.09±1.12, *P*=0.06 |
| **Day 4** | 0.92±0.81, *P*=0.26 | -2.66±2.83, *P*=0.35 | 0.89±0.87, *P*=0.31 |
| **Day 5** | 0.55±0.69, *P*=0.43 | -3.73±3.32, *P*=0.26 | 1.32±0.72, *P*=0.07 |
| **Day 6** | 1.14±0.64, *P*=0.08 | 0.55±1.15, *P*=0.63 | 0.35±0.71, *P*=0.62 |
| **Day 7** | 1.69±0.66, ***P*=0.01** | 1.65±0.98, *P*=0.09 | 0.26±0.72, *P*=0.71 |
| **Day 8** | 2.07±0.66, ***P*=0.002** | 2.19±0.95, ***P*=0.02** | -0.38±0.72, *P*=0.60 |
| **Day 9** | 2.05±0.78, ***P*=0.009** | 1.31±1.14, *P*=0.25 | -0.33±0.82, *P*=0.69 |
| **Day 10** | 1.08±0.72, *P*=0.14 | 2.54±1.28, *P*=0.05 | -1.14±0.92, *P*=0.12 |

*Models are presented as β ± standard deviation. Abbreviations: modified Rankin Scale (mRS), delayed cerebral ischemia (DCI)

**Supplementary Table 13:** Demographics and baseline characteristics based on high or low day 8 eosinophil count.

|  | **Total** | **Low Day 8 Eosinophils^#^** | **High Day 8 Eosinophils** | ***P*** |
| --- | --- | --- | --- | --- |
| **N** | 239 | 165 | 74 |  |
| **Age Median (years)^*^** | 52 (45, 61) | 52 (45, 62) | 51 (46, 59) | 0.97 |
| **Sex (female)**^†^ | 164 (68.6) | 114 (69.1) | 50 (67.6) | 0.933 |
| **HHS 4-5**^†^ | 142 (59.4) | 97 (58.8) | 45 (60.8) | 0.0503 |
| **mFS 3-4**^†^ | 184 (77.0) | 137 (83.0) | 47 (63.5) | **0.002** |
| **GCE**^†^ | 32 (13.4) | 18 (10.9) | 14 (18.9) | 0.14 |
| **IVH**^†^ | 151 (63.2) | 107 (64.8) | 44 (59.5) | 0.513 |
| **Outcomes** |  |  |  |  |
| **Mortality**^†^ | 10 | 6 (3.6) | 4 (5.4) | 0.778 |
| **Hospital LOS^*^** | 15 (11, 21) | 15 (11, 20) | 15 (12, 22) | 0.598 |
| **ICU LOS^*^** | 11 (9, 15.5) | 11 (9, 15) | 12 (9, 16) | 0.644 |
| **Presence of infection**^†^ | 74 (31.0) | 49 (29.7) | 25 (33.8) | 0.631 |
| **DCI**^†^ | 58 (24.3) | 42 (25.5) | 16 (21.6) | 0.634 |
| **Poor discharge mRS** | 78 (32.6) | 46 (27.9) | 32 (43.2) | 0.028 |
| **Angiographic VS**^†^ | 98 (41.0) | 67 (40.6) | 30 (41.9) | 0.999 |
| **EVD Days^*^** | 9 (8, 12.8) | 9 (8, 12) | 10 (8, 13) | 0.081 |
| **VPS**^†^ | 160 (66.9) | 117 (70.9) | 43 (58.1) | 0.072 |

Data are presented as *median (interquartile range) or ^†^N (percent). ^#^Eosinophils are dichotomized as low (<0.2 x 10^3^ cells/μL) or high (≥0.2 x 10^3^ cells/μL). *P*-values that are statistically significant are in bold. Abbreviations: modified Rankin (mRS), Global Cerebral Edema (GCE), Intraventricular Hemorrhage (IVH), Hunt Hess Score (HHS), modified Fisher Scale (mFS), external ventricular drain (EVD), intensive care unit (ICU), vasospasm (VS), length of stay (LOS), delayed cerebral ischemia (DCI), ventriculoperitoneal shunt (VPS)
